# Effects of Mifepristone on Nonalcoholic Fatty Liver Disease in a Patient with a Cortisol-Secreting Adrenal Adenoma

**DOI:** 10.1155/2017/6161348

**Published:** 2017-11-19

**Authors:** Enzo Ragucci, Dat Nguyen, Michele Lamerson, Andreas G. Moraitis

**Affiliations:** ^1^Diabetes and Endocrinology Consultants, 2 Crosfield Ave, No. 204, West Nyack, NY 10994, USA; ^2^Corcept Therapeutics, 149 Commonwealth Drive, Menlo Park, CA 94025, USA

## Abstract

Cushing syndrome (CS), a complex, multisystemic condition resulting from prolonged exposure to cortisol, is frequently associated with nonalcoholic fatty liver disease (NAFLD). In patients with adrenal adenoma(s) and NAFLD, it is essential to rule out coexisting endocrine disorders like CS, so that the underlying condition can be properly addressed. We report a case of a 49-year-old woman with a history of hypertension, prediabetes, dyslipidemia, biopsy-confirmed steatohepatitis, and benign adrenal adenoma, who was referred for endocrine work-up for persistent weight gain. Overt Cushing features were absent. Biochemical evaluation revealed nonsuppressed cortisol on multiple 1-mg dexamethasone suppression tests, suppressed adrenocorticotropic hormone, and low dehydroepiandrosterone sulfate. The patient initially declined surgery and was treated with mifepristone, a competitive glucocorticoid receptor antagonist. In addition to improvements in weight and hypertension, substantial reductions in her liver enzymes were noted, with complete normalization by 20 weeks of therapy. This case suggests that autonomous cortisol secretion from adrenal adenoma(s) could contribute to the metabolic and liver abnormalities in patients with NAFLD. In conclusion, successful management of CS with mifepristone led to marked improvement in the liver enzymes of a patient with long-standing NAFLD.

## 1. Introduction

Nonalcoholic fatty liver disease (NAFLD) is a common cause of chronic liver disease in westernized countries, affecting 17% to 30% of the population [1]. Defined histologically as the accumulation of fat within the hepatocytes that exceeds 5% of liver weight [1, 2], NAFLD is generally considered the hepatic manifestation of metabolic syndrome [3]. Liver damage associated with NAFLD can range from simple steatosis to nonalcoholic steatohepatitis (NASH), which may progress to cirrhosis, liver failure, and hepatocellular carcinoma [1, 2]. NAFLD is strongly associated with risk factors including obesity, insulin resistance, type 2 diabetes, hypertension, and dyslipidemia. Although secondary causes of NAFLD (e.g., lipid metabolism disorders, medications, and other diseases) occur in the minority of cases [1], it is important to exclude them during the differential diagnosis, especially in patients with coexisting adrenal adenoma(s).

Both hypercortisolism from exogenous sources and that from endogenous sources are recognized causes of NAFLD [3]. For instance, NAFLD is frequently seen in patients with Cushing syndrome (CS) [4], a complex, multisystemic condition resulting from prolonged exposure to cortisol. A computed tomography- (CT-) based study found hepatic steatosis in 20% of CS patients with active disease [4].

The mechanisms by which cortisol impacts lipid metabolism are complex [5, 6] and not yet fully elucidated. Here we report a case of autonomous cortisol secretion due to an adrenal adenoma in which medical therapy with the competitive glucocorticoid receptor antagonist mifepristone resulted in biochemical remission of NAFLD.

## 2. Case Presentation

**Figure 1:**
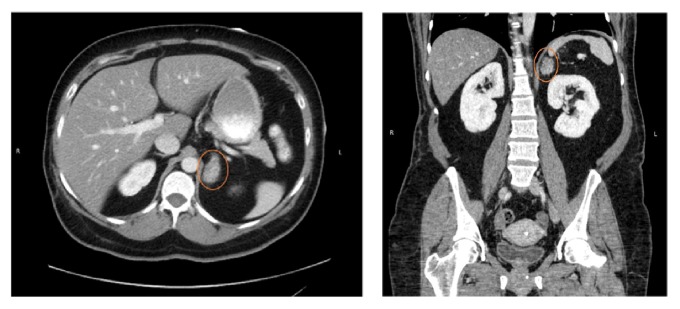
Images showing left adrenal adenoma measuring 2.9 × 1.9 × 2.5 cm (shown in circles).

A 49-year-old woman with a medical history of hypertension, prediabetes, dyslipidemia, and histologically confirmed NASH (80% diffused steatosis with pericellular inflammation) was referred by her primary care provider for endocrine evaluation for complaint of persistent weight gain with central obesity. Two years prior to the referral she was discovered to have a left adrenal adenoma on a routine abdominal CT scan for evaluation of nephrolithiasis. Phenotypic features of overt CS (i.e., moon face, striae, buffalo hump, etc.) were not present, and biochemical evaluation of the adenoma was not performed at that time. At referral, the patient still did not have overt phenotypic features of CS. A follow-up scan revealed a stable benign left adrenal adenoma (2.9 × 1.9 × 2.5 cm) measuring 14 Hounsfield units on noncontrast CT (Figure 1). The adenoma was described as lobulated with a high fat content. Baseline clinical characteristics and laboratory findings are listed in Table 1.

Results of hormonal testing were negative for pheochromocytoma and primary aldosteronism. Testing for autonomous cortisol secretion showed mildly elevated urinary free cortisol (UFC), failure to suppress cortisol on multiple 1-mg overnight dexamethasone suppression tests (DSTs), elevated late-night salivary cortisol, suppressed adrenocorticotropic hormone (ACTH) on multiple tests, and low dehydroepiandrosterone sulfate (DHEA-S) (Table 1).

**Figure 2:**
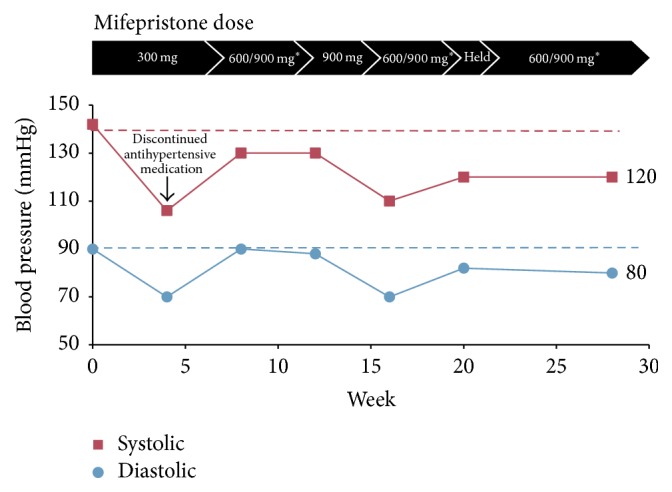
Change in blood pressure over time after initiating mifepristone treatment.  ^*∗*^Alternating daily doses of 600 and 900 mg.

**Figure 3:**
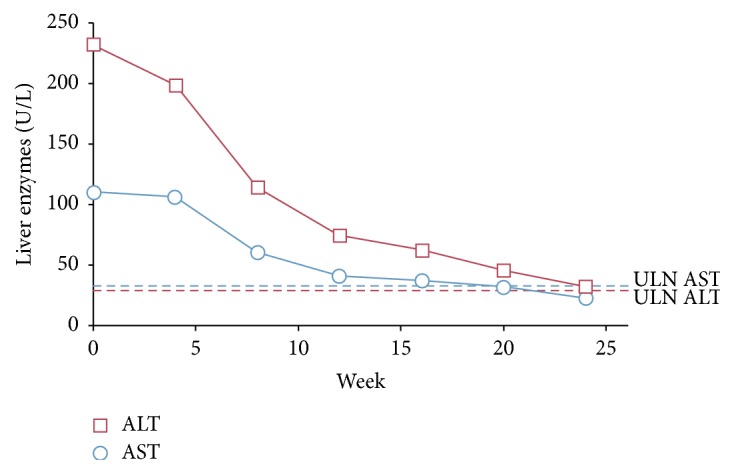
Change in liver enzymes over time after initiating mifepristone treatment. ALT, alanine transaminase; AST, aspartate transaminase; ULN, upper limit of normal.

The patient declined adrenalectomy. Ketoconazole was not considered because of the patient's fatty liver and elevated liver enzymes. Medical therapy with mifepristone (Korlym®, Corcept Therapeutics, Menlo Park, CA) was initiated at 300 mg per day and increased to 900 mg during the 34 weeks of treatment. After 4 weeks, her antihypertensive medication (amlodipine/olmesartan medoxomil 10/20 mg) was discontinued and her blood pressure remained stable (Figure 2). During the course of mifepristone treatment, she lost 16 lbs and marked improvement in her liver enzymes was noted, with complete normalization by week 20 (Figure 3).

At week 20 she complained of vaginal spotting, and a pelvic ultrasound showed multiple uterine fibroids. Subsequently she elected to undergo a hysterectomy and a left adrenalectomy. Mifepristone was discontinued 2 weeks prior to the adrenalectomy. Her prediabetes remained stable during mifepristone treatment; HbA1c was 40 mmol/mol before surgery.

**Figure 4:**
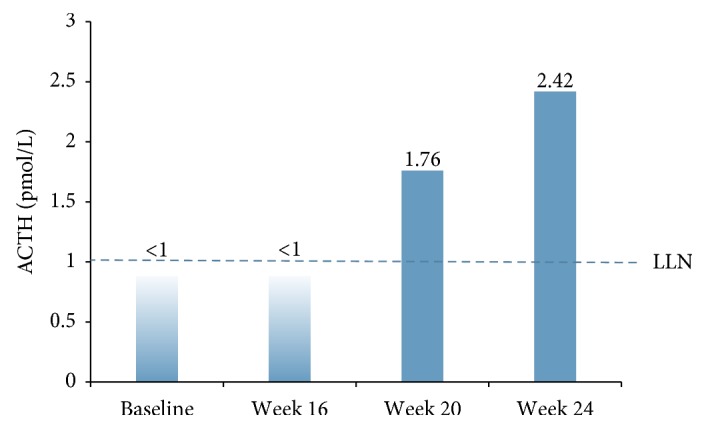
Change in ACTH over time after initiating mifepristone treatment. ACTH, adrenocorticotropic hormone; LLN, lower limit of normal.

During the 34-week course of medical therapy with mifepristone, the patient's hypothalamic-pituitary-adrenal (HPA) axis recovered, as indicated by increases in ACTH levels from undetectable at baseline and week 16 to 2.4 pmol/L at week 24 (Figure 4). She was treated postoperatively with glucocorticoid replacement (hydrocortisone 50 mg), which was tapered and discontinued within 6 weeks. Her liver enzymes remained within the normal range and there were no further changes in blood pressure and weight 1 year postoperatively. Her most recent HbA1c was 33 mmol/mol (5.2%).

## 3. Discussion

We have described a case of hypercortisolism due to an adrenal adenoma associated with biopsy-confirmed NASH. The patient was treated medically with mifepristone therapy for 34 weeks. In addition to improvement in cardiometabolic parameters associated with hypercortisolism, we also noted marked improvement in her liver function tests (LFTs) during treatment.

NAFLD is associated with cardiometabolic risk factors (obesity, diabetes, dyslipidemia, and hypertension) and is diagnosed by hepatic steatosis on imaging or histology and exclusion of other causes of liver disease [7]. Although LFTs are a good surrogate biomarker for NAFLD, a substantial portion of patients, 79% according to one study [8], may have normal LFTs. Thus, liver biopsy, while being costly and invasive, remains the only definitive method for diagnosis.

NAFLD is frequently seen in patients with CS [4], which is not surprising as metabolic derangements are also common in CS. In fact, one study found a higher prevalence and greater severity of NAFLD among patients with CS when compared to matched cohorts of nonfunctioning adrenal adenomas and controls [9]. A high prevalence of metabolic syndrome features and cardiometabolic risk factors has also been reported in patients with adrenal adenoma(s) [10–12]. Other studies have shown HPA axis dysfunction among patients with NAFLD [13–15]. In overweight/obese NAFLD patients, HPA axis dysfunction was correlated with the severity of NAFLD [16]. Together, these studies suggest that subtle and chronic activation of the HPA axis can contribute to the development and progression of NAFLD.

Hypercortisolism without classically described features of overt CS occurs in up to 30% of patients with adrenal adenomas [17]. Clinical assessment for signs and symptoms of hypercortisolism, as well as biochemical evaluation using the 1-mg overnight DST, is recommended as part of the work-up for patients with newly discovered adrenal adenomas [18]. In our case, the patient was not referred for endocrine evaluation until 2 years after the adenoma was discovered and her comorbidities had worsened. This case underscores the need for greater awareness among primary care providers of the importance of cortisol evaluation for benign adrenal adenomas, regardless of clinical symptomology.

Mifepristone, a competitive glucocorticoid receptor antagonist, is an effective medical therapy for patients with endogenous CS [19]. Patients from the SEISMIC trial demonstrated significant improvement in glucose abnormalities (assessed by oral glucose tolerance test), significant reductions in fasting plasma glucose and HbA1c, and a rapid and significant decrease in mean area under the curve insulin levels [19]. Together, these suggest improved insulin sensitivity.

The effects of mifepristone on metabolic parameters in patient populations outside of CS have also been explored. In a small study of patients with type 2 diabetes and fatty liver, short-term doses of mifepristone and metyrapone, a cortisol biosynthesis inhibitor, appeared to improve insulin sensitivity [10]. Two other clinical studies conducted in healthy men demonstrated that mifepristone significantly attenuated not only the side effect of weight gain caused by second-generation antipsychotic medications, but also the increases in fasting plasma insulin and triglyceride levels caused by their use [20, 21].

In our patient with hypercortisolism and biopsy-confirmed NASH, mifepristone treatment was associated with dramatic improvement in LFTs, as well as improvements in weight and hypertension leading to discontinuation of her antihypertensive medication. Follow-up liver imaging was not available for comparison; however, the overall improved functional and metabolic status of the patient, along with no signs or symptoms of deteriorating liver function (cirrhosis), support the LFT findings. The effect of mifepristone on LFTs was a novel finding and suggests that mifepristone may offer an alternative treatment to surgery in patients with hypercortisolism and NAFLD. It also may provide a useful method to assess the contribution of autonomous cortisol secretion of an adrenal adenoma to the cardiometabolic profile and liver abnormalities in patients with NAFLD. Larger prospective studies are needed to determine whether mifepristone use in patients with NAFLD and adrenal adenoma(s) can be used as a screening tool to select patients who will benefit from adrenalectomy.

## Figures and Tables

**Table 1:** Baseline patient characteristics and laboratory findings.

Parameter	Result
Age, years	49
BMI, kg/m^2^	30.6
BP, mmHg	142/90
HbA1c, mmol/mol	41
Lipids, mmol/L	
Total cholesterol	7.1
LDL	5.3
TG	1.5
Liver function	
AST, U/L (normal 2–40)	110
ALT, U/L (normal 2–60)	232
Endocrine evaluation	
UFC, nmol/d (normal 11–138)	172.8
1-mg DST, nmol/L (normal < 49.5)	364.3; 345.0
Late night salivary cortisol, nmol/L (normal < 2.5)	5.2
ACTH, pmol/L (normal 1.1–9.9)	<1.1 × 2
DHEA-S, *μ*mol/L (normal 1.1–7.9)	0.54

ACTH, adrenocorticotropic hormone; ALT, alanine transaminase; AST, aspartate transaminase; BMI, body mass index; BP, blood pressure; DHEA-S, dehydroepiandrosterone sulfate; DST, dexamethasone suppression test; HbA1c, glycated hemoglobin A1c; LDL, low-density lipoprotein; TG, triglyceride; UFC, urinary free cortisol.
